# Worldwide variations in *EGFR *somatic mutations: a challenge for personalized medicine

**DOI:** 10.1186/1746-1596-7-13

**Published:** 2012-02-01

**Authors:** Pierre-Jean Lamy, William Jacot

**Affiliations:** 1 Laboratoire de Biologie Spécialisée et Oncogénétique, CRLC Val d'Aurelle-Paul Lamarque, 208, rue des Apothicaires, F-34298 France; 2 Département d'Oncologie Médicale, CRLC Val d'Aurelle-Paul Lamarque, 208, rue des Apothicaires, F-34298 France

**Keywords:** Breast Cancer, EGFR, activating mutations, triple negative, geographic variations

## Abstract

Two studies recently reported around 10% of *EGFR *activating mutations in triple negative breast cancers from Asian patients. However, we did not find any EGFR activating mutation in a series of 229 breast tumor samples from European patients. Like in lung cancer, the *EGFR *mutation profiles seem to be related to the ethnical origin of patients. This is an important point that should be considered when developing anti-EGFR therapies.

## Commentary

We have read with great interest the study on Epidermal Growth Factor Receptor (EGFR) mutations in breast carcinoma by Ning Lv et al published in Diagnostic Pathology [1]. Since in lung cancer the choice of anti-EGFR therapy is based on the presence of *EGFR *activating mutations, and due to the importance to the EGFR pathway in breast cancer, it is crucial to evaluate the presence of these mutations in the different subtypes of breast cancer. The authors reported that, in their series of 139 breast cancers, two (1.4%) tumors harbored activating *EGFR *mutations in exon 19 and 21 of this gene. One tumor was classified as estrogen receptor (ER) (1/105, 0.8%) positive and the other was a triple negative (TN) tumor (1/10, 10%). Similarly, in a recent study on 70 TN breast cancers, which were previously described to often present an EGFR addiction, Teng et al. found 8 (11.4%) activating *EGFR *mutations in exon 19 and 21 [2]. Both works reported findings that were obtained using tumor samples from Asian patients. We recently analyzed a series of 229 TN tumor samples from European patients with primary breast cancer and found that none had activating *EGFR *mutations [3]. In previous reports concerning smaller series of Caucasians patients, no *EGFR *activating mutation was found whatever the subtype of breast cancer studied [4-6]. Similarly, in a cohort of 58 Japanese patients, Toyama et al failed to find exon 19 and 21 *EGFR *mutations [7]. These results underline geographic differences in the presence of *EGFR *activating mutations as it was already described for non-small cell lung cancer [8]. This mutational event, which appears to be mostly limited to Chinese patients with TN breast cancer, could be related to ethnic or environmental factors. However, the question of the origin of the geographical variations in the occurrence of *EGFR *mutations in lung and breast cancers is unresolved.

Interestingly, a case of breast metastasis from a primary lung cancer that was confirmed by the detection of the same *EGFR *mutation has been recently reported [9]. This observation confirms that the presence of this mutation is not an obstacle to metastasization/tumor cell growth in the breast microenvironment. Inversely, we should not forget that some breast cancer characteristics, like ER expression and HER2 over-expression, have been also described in lung cancers [10,11]. Lung and breast cancer subtypes seem to share some biomarkers and thus could respond to the same therapies.

At the time of personalized medicine in oncology, more than ever, molecular patterns break the limits of classifying tumors according to their localization. Mutation profiles have to be analyzed in sub-groups of specific populations. This is a new and promising challenge for the design of clinical trials involving targeted therapies.

## Competing interests

The authors declare that they have no competing interests.

## Authors' contributions

PJL has been involved in drafting the manuscript. WJ has been involved in the critical revision of the manuscript intellectual content. All authors read and approved the final manuscript.
